# Morphea Profunda with Tertiary Lymphoid Follicles: Description of Two Cases and Review of the Literature

**DOI:** 10.3390/dermatopathology9010003

**Published:** 2022-01-10

**Authors:** Angelo Cassisa, Margherita Vannucchi

**Affiliations:** 1 Section of Pathology, Department of Oncology, San Giovanni di Dio Hospital, USL Centro Toscana, 50143 Florence, Italy; 2 Section of Pathology, Department of Medical Biotechnology, University of Siena, 53100 Siena, Italy; margherita.van@gmail.com

**Keywords:** morphea, morphea profunda, fibrosis, lymphoid hyperplasia, tertiary lymphoid tissue

## Abstract

Morphea profunda or subcutaneous (deep) morphea is a variant of localized morphea, characterized by one or more ill-defined, deep sclerotic plaque. Preferential sites are the abdomen, trunk, sacral area, or extremities. The presence of hyperplastic lymphoid follicles in the context of the sclerotic bands of morphea is rarely described. Localized scleroderma is sustained by a profibrotic inflammatory profile. Transforming growth factor-β (TGF-β), an imbalance between functional subclasses of T-lymphocytes (innate immune cells) has a role in activate collagen deposition. In this case report, we present two cases of morphea profunda with lymphoid follicular hyperplasia. A systematic review of the literature on the pathophysiology of localized scleroderma is also presented, with particular reference to the presence of lymphoid structures.

## 1. Introduction

Scleroderma is a spectrum of conditions characterized by the deposition of collagen. Plaque morphea is a localized form, presenting as solitary or a few indurated plaques, most often localized on the abdomen or extremities.

Lesions usually progress slowly or remain stable for years.

The term ‘morphea profunda’ is used to include cases with variable deep dermal, subcutaneous, and fascial involvement. Infections, trauma, radiation or drugs can potentially trigger the disease. Pro-fibrotic mediators, such as transforming growth factor-beta (TGF-beta), drive the fibrotic process. Lymphoid infiltrates are a common finding in cutaneous lesions of connective tissue diseases but are anecdotally reported in scleroderma.

We report two cases of ‘morphea profunda’ with a rare finding—an association with tertiary lymphoid follicles.

We performed a comprehensive search of the existing literature on ‘morphea profunda’ “tertiary lymphoid follicles” using the PubMed Database and reviewed the previously reported cases.

## 2. Case Report

### 2.1. Case 1

A seventy-five-year-old woman came to our attention because of a dyschromic, hard-elastic, roughly triangular skin plaque that arose on the left leg over one year and gradually hardened. A previous application of topical steroid therapy had proved useless.

The patient was studied using echo-color doppler of the lower limbs, providing evidence of a normal deep and superficial venous circulation.

Blood tests, serology for Borrelia burgdorferi infection, anti-nuclear antibodies (ANA), extractable nuclear antigens (ENA) and other inflammation indicators were negative.

Therefore, a skin biopsy was taken.

The histopathological analysis showed dermo-hypodermic fibrous thickening. In particular, the hypodermic adipose tissue was largely replaced by broad sclerotic bands with a transverse orientation.

The thickening of collagen bundles and the disorganization of the elastic fibers were highlighted using Weigert staining, sustaining a diagnosis of morphea profunda.

**Figure 1:**
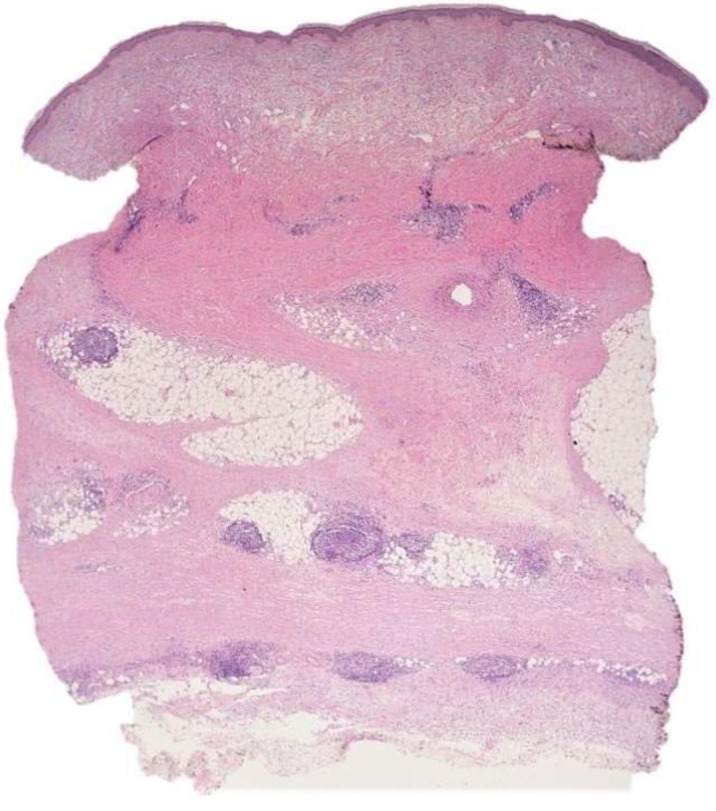
Case 1: Lympho-plasmacellular nodular infiltrate between broad sclerotic bands with transverse orientation replacing dermo-hypodermal tissue.

**Figure 2:**
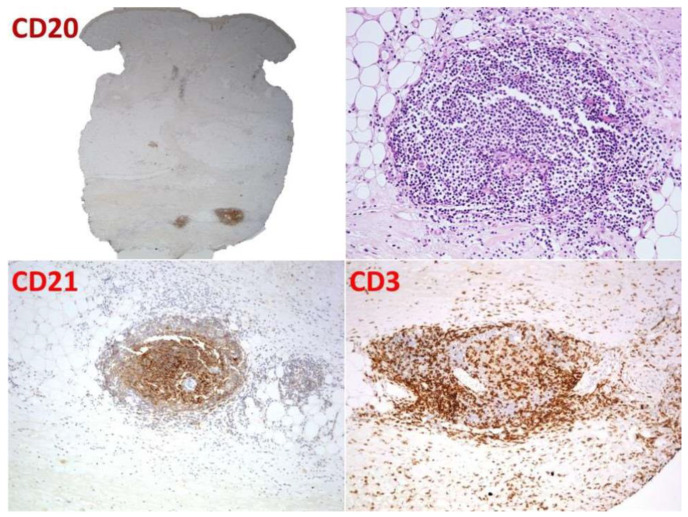
Case 1: Follicular organization underlined by CD21-positive meshwork pattern of follicular dendritic cells.

Between the sclerotic bundles, a lympho-plasmacellular nodular infiltrate was present (Figure 1). The follicular organization was underlined by a CD21-positive meshwork pattern of follicular dendritic cells. Thin mantle zones surrounded reactive germinal centers (Figure 2). CISH for light chains demonstrated no restriction in the plasma cell component.

The focal aspects of the membrano-cystic degeneration of the adipose tissue with sporadic multinucleated histiocytes were an associated finding.

The patient was treated with topical steroids. The plaque was stable after 12 months.

### 2.2. Case 2

A 48-year-old woman without any past medical history came to our attention due to skin hardening on her right leg, below the knee.

The lesion was dyschromic and depressed with irregular limits.

An ultrasound examination demonstrated dermo-hypodermic fibrous thickening with features of chronic oedema. The peroneal long muscle and the superficial peroneal muscle showed a diffuse interstitial oedema, interpreted as unresolved myositis.

The patient was receiving therapy with methotrexate.

**Figure 3:**
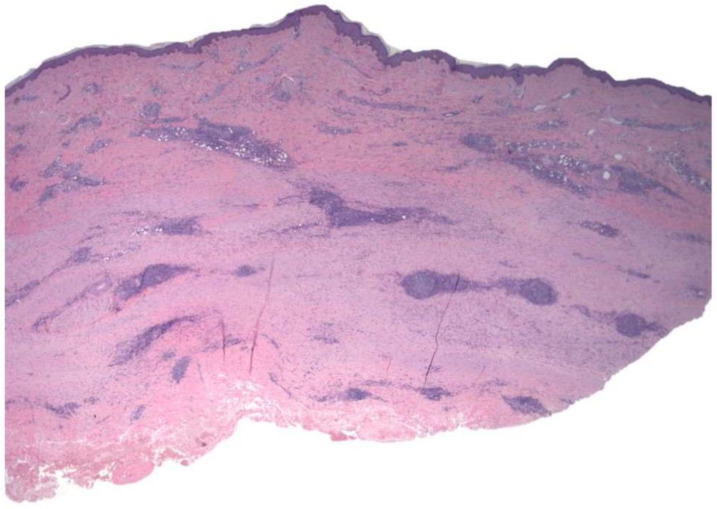
Case 2: Full-thickness sclerosis of the dermal and hypodermic component, including B lymphoid nodules.

**Figure 4:**
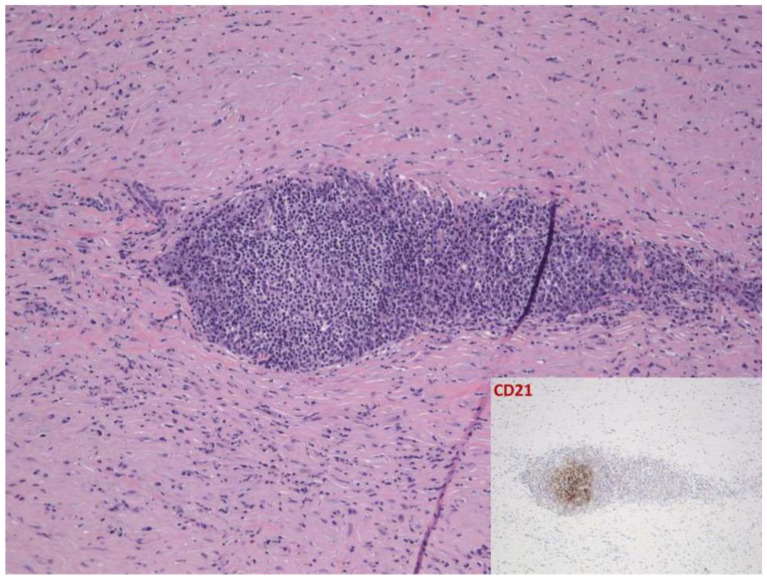
Case 2: Follicular architecture underlined by dendritic cell meshwork.

Histological examination showed full-thickness hardening of the dermal and hypodermic components with broad sclerotic bundles with a transversal orientation and lymphoid nodules sandwiched between them (Figure 3). A follicular organization with a dendritic cell meshwork was documented (Figure 4). The biopsy did not include the fascia and underlying muscle. Again, a diagnosis of morphea profunda was made.

Topical steroid therapy did not significantly improve the clinical aspect. The patient was lost to follow-up.

## 3. Discussion

In scleroderma, an excess of collagen synthesis takes place. The presence of an inflammatory infiltrate defines an active phase, followed by a fibrotic one.

In deep morphea, the fibrotic process involves the deep dermis and hypodermis. Some authors prefer the term subcutaneous morphea when the involvement of subcutaneous fat overwhelms dermal involvement. Fibrotic bands parallel to the skin surface substitute adipose lobules, thereby entrapping residual adipocytes [1,2]. 

The increased collagen synthesis in morphea is mainly determined by the stimulation of dermal fibroblasts. The activation of myofibroblast progenitors is a response to paracrine signals, the best known of which is TGF-β. TGF-β is produced by a wide range of inflammatory cells, endothelial cells, and epithelial cell types. Unlike other cytokines, it is also present in latent form (L-TGF-β 1) bound to the ECM (extracellular matrix). In physiological conditions, TGF-β1 regulates the healing process, remodeling the ECM with impacts on every cell type involved [3]. Proteases like plasmin and thrombin, as well as ROS, can quickly activate L-TGF-β 1 from the extracellular reservoir [4]. 

TGF-beta dysregulation can enhance the formation of the extracellular matrix. Specifically, in localized scleroderma, high levels of TGF-β and latent TGF-β-binding protein 4 have been documented by immunohistochemistry measurements in sclerotic skin tissue [4].

Grabell [5], in his case series, postulated that traumatism could act as a trigger in localized morphea, and the involvement of TGF-β activation in tissue repair may sustain this hypothesis. The extent of fibrosis in systemic sclerosis also correlates with the activation of dermal innate lymphoid cells [6,7]; however, no specific data are available regarding their role in localized scleroderma.

Inflammatory infiltrate in localized scleroderma is more marked in early lesions than in late lesions. The number of lymphocytes, macrophages and plasma cells varies broadly, and they have an interstitial and or perivascular distribution. In cases with involvement of the fascia by eosinophils, eosinophilic fasciitis must be considered in differential diagnoses.

The presence of tertiary lymphoid follicles is rarely reported in scleroderma [8,9,10]. Whitaker [10] described lymphoid aggregates with germinal center formation in three patients out of a series of five localized cases of morphea profunda localized on the trunk. None of these cases showed progression or significant clinical distinction from the others. Elsewhere, a case of linear morphea [9] was associated with Grave’s disease. Lymphoid hyperplasia with germinal center formation was documented in pre-existing morphea plaques after methotrexate administration [11]. Tertiary lymphoid follicles are frequently described in autoimmune/autoinflammatory conditions [12]. In lupus profundus, lymphoid nodules are associated with diagnostic clues, i.e., mucin deposition, periadnexal infiltrate, junctional infiltrate, and CD4+ lymphocytes rimming fat cells. Lupus profundus overlapping with morphea was described by Marzano et al. [13]. Moreover, concurrent lesions of lupus and morphea were described by other authors [14,15,16].

Inflamed tissues in rheumatoid arthritis, Sjögren’s syndrome, multiple sclerosis and myasthenia gravis often harbor lymphoid follicles. In all of these autoimmune conditions, a germinal center response provides oligoclonal, autoreactive plasma cells that are probably involved in tissue damage or remodeling [17,18]. Circulating autoantibodies have also been documented in morphea, testifying to the significant activation of the immune system [19,20].

The development and assembly of lymphoid follicles requires a complex mix of homeostatic lymphoid chemokines. In this regard, TGF-β seems to play a central role beyond its fibrogenetic one. TGF-β signaling is involved in CXCL13-producing CD4(+) T cells lacking Tfh-cell feature differentiation [21,22,23]. CXCL13, as well as being a profibrogenic factor, recruits B lymphocytes and can drive the mounting of a follicular structure [23]. Germinal center homeostasis and function are therefore maintained by TGF-β signaling [24].

In conclusion, tertiary lymphoid structures are a rare finding in morphea profunda. TGF-β is involved both in fibrogenic processes and in inducing and maintaining tertiary lymphoid follicles. The roles of the local production of immunocompetent cells and antibodies in morphea need to be fully explained. Consistent with data from the literature, the two cases described did not progress to systemic disease.
